# Investigating CXCR4 expression of tumor cells and the vascular compartment: A multimodal approach

**DOI:** 10.1371/journal.pone.0260186

**Published:** 2021-11-18

**Authors:** Marta Braga, Chee Hau Leow, Javier Hernandez Gil, Jin H. Teh, Laurence Carroll, Nicholas J. Long, Meng-Xing Tang, Eric O. Aboagye

**Affiliations:** 1 Department of Surgery and Cancer, Faculty of Medicine, Imperial College London, London, United Kingdom; 2 Department of Bioengineering, Faculty of Engineering, Imperial College London, London, United Kingdom; 3 Department of Chemistry, Faculty of Natural Sciences, Imperial College London, London, United Kingdom; Indiana University Purdue University at Indianapolis, UNITED STATES

## Abstract

The C-X-C chemokine receptor 4 (CXCR4) is G protein-coupled receptor that upon binding to its cognate ligand, can lead to tumor progression. Several CXCR4-targeted therapies are currently under investigation, and with it comes the need for imaging agents capable of accurate depiction of CXCR4 for therapeutic stratification and monitoring. PET agents enjoy the most success, but more cost-effective and radiation-free approaches such as ultrasound (US) imaging could represent an attractive alternative. In this work, we developed a targeted microbubble (MB) for imaging of vascular CXCR4 expression in cancer. A CXCR4-targeted MB was developed through incorporation of the T140 peptide into the MB shell. Binding properties of the T140-MB and control, non-targeted MB (NT-MB) were evaluated in MDA-MB-231 cells where CXCR4 expression was knocked-down (via shRNA) through optical imaging, and in the lymphoma tumor models U2932 and SuDHL8 (high and low CXCR4 expression, respectively) by US imaging. PET imaging of [^18^F]MCFB, a tumor-penetrating CXCR4-targeted small molecule, was used to provide whole-tumor CXCR4 readouts. CXCR4 expression and microvessel density were performed by immunohistochemistry analysis and western blot. T140-MB were formed with similar properties to NT-MB and accumulated sensitively and specifically in cells according to their CXCR4 expression. In NOD SCID mice, T140-MB persisted longer in tumors than NT-MB, indicative of target interaction, but showed no difference between U2932 and SuDHL8. In contrast, PET imaging with [^18^F]MCFB showed a marked difference in tumor uptake at 40–60 min post-injection between the two tumor models (p<0.05). *Ex vivo* analysis revealed that the large differences in CXCR4 expression between the two models are not reflected in the vascular compartment, where the MB are restricted; in fact, microvessel density and CXCR4 expression in the vasculature was comparable between U2932 and SuDHL8 tumors. In conclusion, we successfully developed a T140-MB that can be used for imaging CXCR4 expression in the tumor vasculature.

## Introduction

The C-X-C chemokine receptor 4 (CXCR4, also known as fusin and CD184) is a seven-transmembrane domain G protein-coupled receptor (GPCR). Upon binding its natural ligand, the CXCL12 chemokine (also known as stromal cell-derived factor-1α, SDF-1α), CXCR4 activates several downstream signaling pathways leading to altered gene expression, migration, survival and proliferation [1]. These pathways are exploited during tumorigenesis, especially in the metastatic process, where CXCR4-expressing tumor cells are chemotactically homed to organs with abundant levels of CXCL12—such as the liver, bone marrow, lungs and lymph nodes [2–4]. Indeed, CXCR4 overexpression has been shown in over 20 human tumor types [5–10], and correlated with tumor aggressiveness and higher probability of recurrence [11–16].

This receptor has been gaining attention as a therapeutic target [17]. Several antagonists have been developed to date, with the most important falling into two main classes of compounds: peptide-based—such as the T140 peptide [18] and its derivatives [19]—or small-molecules–such as AMD3100 [20, 21] and AMD3465 [22]. Imaging agents analogous to these antagonists have also been developed, as sensitive depiction of CXCR4 could be used to effectively stratify patients [23] and monitor therapeutic outcome with emphasis on SPECT imaging [24–26], optical imaging [27–32] and PET [33–44]; PET-dedicated radiotracers have seen the most success, with [^68^Ga]Pentixafor, a peptide-based CXCR4-targeting molecule, currently in multiple clinical trials including patients with neuroendocrine tumors (*ClinicalTrials*.*gov identifier*: *NCT03335670*), multiple myeloma and lymphoma (*ClinicalTrials*.*gov identifier*: *NCT03436342*). Despite the relative success of these PET agents, their clinical use is limited by important considerations such as radiation dose, high cost, and relative inaccessibility of PET scanners.

Ultrasound (US) has been gaining momentum as an alternative imaging technique due to its cost-effectiveness, portability, widespread availability and safety. Although its applications have been mainly restricted to anatomical and functional imaging, the advent of US contrast agents–microbubbles (MB)–and their functionalization (i.e. addition of a targeting ligand to their lipid shell leading to adhesion to specific molecular targets) have made molecular imaging possible with US. Moreover, the use of MB enables imaging with unprecedented sensitivity, and the lack of ionizing radiation makes this modality apt for routine clinical use such as screening and therapeutic monitoring. Due to their micron-scale size, MB are restricted to the vascular compartment and are therefore particularly well suited for imaging disease processes that impact expression of vascular markers, such as inflammation or angiogenesis [45]. Although the role of CXCR4 is better characterized in the tumor interstitium, the CXCR4/CXCL12 axis is also expressed in the tumor vasculature [46, 47] where it is known to promote angiogenesis and vascularization through induction of VEGF [48, 49] and/or recruitment of endothelial progenitor cells [50]. Little is known regarding how CXCR4 expression by the tumor cells affects the vasculature, and it would be interesting if CXCR4 is sufficiently expressed in the tumor vasculature to be detected with the easily-accessible US. Thus, in this work, we developed a CXCR4-targeted MB by functionalizing its shell with the CXCR4-specific T140 peptide and hypothesized that it would produce contrast enhancement proportionally to receptor expression in the vasculature. Furthermore, we investigated how vascular CXCR4 reflects whole-tumor expression through comparison with imaging data produced by a tumor-penetrating CXCR4-dedicated PET tracer, [^18^F]MCFB [33].

## Material and methods

All reagents were purchased from Sigma-Aldrich (Dorset, UK) unless otherwise specified.

### Synthesis of Lipo-PEG-T140

**Fig 1:**
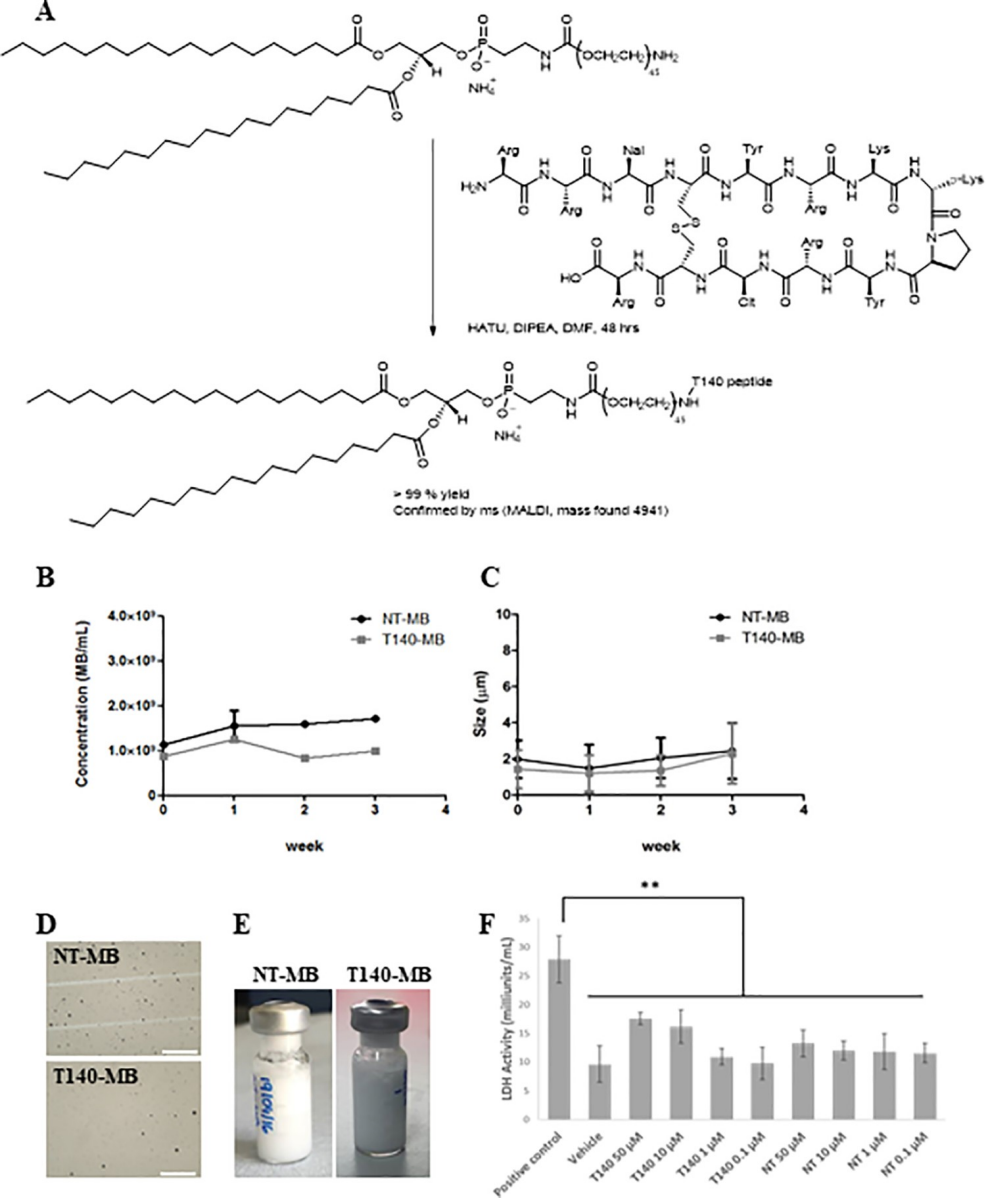
Development of a CXCR4-targeted microbubble. (A) Scheme of synthesis of DSPE-PEG_2000_-T140 lipo-PEG-peptide through covalent coupling of the TN14003 peptide with the DSPE-PEG_2000_-NH_2_ lipid. (B) Lipid composition of NT-MB and T140-MB expressed in mole fraction (%). Microbubble concentration (C) and size (D) were determined using dedicated software for analysis of optically acquired FOV as exemplified in (E). Data represents mean ± SEM, n = 4. Images were obtained under 400x magnification and scale bar represents 100 μm. (F) Typical appearance of NT-MB and T140-MB vials after shaking. (F) Cell membrane integrity analysis by measuring LDH released into the supernatant by HepG2 cells upon treatment with indicated doses of microbubble T140-lipid mix and non-targeted lipid mix for 4 hours. The LDH provided by the manufacturer was used as the positive control. The data are presented as mean ± SD, n = 6; **P < 0.01.

The CXCR4-targeting T140 peptide analogue, TN14003 (referred hitherto as T140) with the sequence Succ-RR-2Nal-(CYRKkPYR-Cit-C)R-NH_2_ was purchased from Cambridge Peptides, Birmingham, UK and coupled to the DSPE-PEG_2000_-NH_2_ lipid by covalent amide bond formation (Fig 1A). Briefly, HATU (1-[Bis(dimethylamino)methylene]-1H-1,2,3-triazolo[4,5-b]pyridinium 3-oxid hexafluorophosphate) was used to generate an active ester from the C-terminal carboxylic acid of T140, and an amide bond was formed with the NH_2_ group of DSPE-PEG_2000_-NH_2_. DSPE-PEG_2000_-NH_2_ (7.9 mg) was dissolved in CHCl_3_ (100 μl) and added to a vial containing TN14003 (5.9 mg) dissolved in DMF (100 μl), DIPEA (0.5 μl) and HATU (1.06 mg). Synthesis was performed stoichiometrically and had a duration time of 25 h, with approximately 1 h of hands-on synthesis and 24 h of stirring at RT. The vial was freeze-dried to evaporate the solvents and stored at– 20°C.

### Microbubble preparation

Both CXCR4-targeted (T140-MB) and non-targeted (NT-MB) MB were produced in-house. The NT-MB’s shell contained a mixture of DPPC, DPPA and DSPE-PEG_2000_-NH_2_ (81.9:8.6:9.5 mole ratio, respectively) (Avanti Lipids, Alabaster, AL, USA) (S1 Table) and in the T140-MB, 4.8% of the DSPE-PEG_2000_-NH_2_ was replaced by the modified lipid, DSPE-PEG_2000_-T140 (Fig 1A and S2 Table). These components were dissolved in CHCl_3_ and aliquoted into 3 mL vials from which the solvent was dried using a N_2_ flow and stored at -20⁰C. Before use, the vials were reconstituted with propylene glycol, phosphate-buffered saline (PBS) and glycerol (16:5:1) whilst stirring. After degassing for a minimum of 15 min, the vial was crimp-sealed and the headspace of the vial was gas-exchanged with 99.0% n-perfluorobutane (FluoroMed L.P., Round Rock, TX, USA). MB were formed via standard mechanical agitation techniques using a Vialmix shaker (Bristol-Myers-Squibb, NY, USA) (2x30 sec shaking). Fluorescent MB were developed by adding 2 μg/mL of Dil stain (1,1’-Dioctadecyl-3,3,3’,3’-Tetramethylindocarbocyanine perchlorate, DilC_18_(3), Thermo Fisher Scientific, Carlsbad, CA USA).

### Optical characterization

Following reconstitution, MB were counted and sized after a dilution in PBS (1:200) and 10 μl were introduced into a haemocytometer, which was allowed to rest for 3 minutes such that MB float to the top of the chamber. A minimum of 10 images of different FOVs were acquired using a 40x UPlanAPO objective lens on an Olympus BX-51 wide-field microscopy UIS2 optical system equipped with a DP70 digital camera. Images were acquired using an Olympus U-RFL-T epifluorescence source and DPController 1.2.1.108 imaging software. Counting and sizing was performed using a dedicated script developed using the MATLAB software (MATLAB 8.2, The Mathworks Inc, Natick, MA, USA).

### Radiosynthesis of [^18^F]MCFB

[^18^F]MCFB was produced following methodology described in Brickute *et al* [33].

### Cell culture

The human diffuse large B cell lymphoma cell lines (DLBCL) SuDHL8 and U2932 were obtained from Dr. Li Jia (Barts Cancer Institute, London, UK), and purchased from Deutsche Sammlung von Mikroorganismen and Zellkulturen GmBH (Braunschweig, Germany), respectively. HepG2 cells were purchased from ATCC (Manassas, USA). CXCR4 expression was modulated in a human triple-negative metastatic breast cancer cell line, MDA-MB-231 (PerkinElmer, Waltham, MA, USA), by using a doxycycline (DOX)-inducible lentiviral pTRIPZ vector encoding for shRNA targeting CXCR4 (pTRIPZ CXCR4 shRNA clone V3THS_346208; Dharmacon, Lafayette, USA) to obtain MDA-MB-231 shCXCR4 or non-targeting shRNA control (Dharmacon) to obtain MDA-MB-231 shSC. Stable clones were FACS sorted based on the expression of a DOX-inducible TurboRFP reporter gene within the same construct. SuDHL8, U2932, MDA-MB-231 shRNA and shSC were cultured in Roswell Park Memorial Institute Medium 1640 (RPMI) and HepG2 was cultured in Dulbecco’s Modified Eagle’s Medium (DMEM). All media was supplemented with 10% Foetal Bovine Serum (FBS), 2mM L-glutamine (Invitrogen™, Thermo Fisher Scientific, Carlsbad, CA USA) and 100 U/mL penicillin-streptomycin (Invitrogen™) in a humidified atmosphere of 5% CO_2_ at 37°C.

### Lactate Dehydrogenase (LDH) activity assay

The cytotoxicity was assessed using a LDH assay which measures the level of plasma membrane damage. The LDH Assay kit was obtained from Sigma-Aldrich®, and was performed using a colorimetric (450 nm) assay that measures NADH reduced by LDH according to the manufacturer’s instructions.

Briefly, the T140-lipid mixture or NT-lipid mixture was dissolved in DMSO (10 μL) and made up to 1 mL with media to achieve a stock concentration of 50 μM T140-lipid, 1% DMSO. This resulted in a fine particulate suspension of lipids, which was diluted to for further use. HepG2 (3000 cells/well) were incubated in 96-well plates for 4 hours with the T140-lipid mixture or NT-lipid mixture at the specified concentrations at a total volume of 100 μL. Plates were allowed to adjust to room temperature, and the 50 μL from each well was collected, centrifuged (5000 *g*, 3 mins), and incubated with 50 μL Master Reaction Mix at 37°C for 15 mins. Background readings were subtracted and the results are expressed as the amount of LDH that catalysed the conversion of lactate into pyruvate to generate 1 μmole of NADH per minute at 37°C. The positive control provided was provided by the manufacturer.

### *In vitro* binding of T140-MB

For *in vitro* MB binding studies, MDA-MB-231 shSC or shCXCR4 cells (1x10^6^) were plated in OptiCell™ plates (VWR, Radnor, Pennsylvania, USA) in 10 mL of media. CXCR4 knockdown was induced by treatment with 0.5 μg/mL of DOX for 24h. Cells were incubated with approximately 1x10^6^ MB/mL (10 mL) of either NT-MB or T140-MB for 15 min at 37°C. During this period, the OptiCell™ plates were flipped to maximize contact between the cells and MB; after incubation, plates were flipped back to the original position and allowed to rest for 5 min before evaluation as to ensure floating of unbound MB to the top of the plate. Blocking studies were performed by incubation with 1 mg/mL of unconjugated T140 for 5 min before and during MB incubation. Fluorescence evaluation of MB binding was carried out as described, albeit with the use of Dil-modified MB. GFP-expressing MDA-MB-231-Fluc-GFP were plated in parallel with these experiments and used as control. Images were acquired using a 40x UPlanAPO objective lens on the Olympus BX-51 wide-field microscope referenced previously.

### Western blot

For evaluation of protein expression, cells (80–90% confluency) were placed on ice, the medium was removed and cells were washed 3 times with ice-cold PBS and lysed with RIPA buffer (Sigma-Aldrich) supplemented with 100X Pierce™ protease and phosphatase inhibitor cocktail (Thermo Fisher Scientific) for 10 min on ice. Excised and snap-frozen tumor tissue samples were homogenized in RIPA lysis buffer supplemented with 100X Pierce™ protease and phosphatase inhibitor cocktail, using the PreCellys 24 homogenizer and CK14 beads-containing tubes (two cycles of 25 seconds at 6500 rpm). Samples were centrifuged at 500 rpm for 5 min at 4°C and supernatant transferred to a new Eppendorf®. Samples were sonicated and total protein concentration was quantified using the Pierce™ BCA protein assay kit (Thermo Fisher Scientific). Lysates were mixed with NuPage® LDS loading buffer and reducing agent (Invitrogen™), and denatured at 70°C for 10 min. Equal amounts of protein (30 μg) were resolved on 4–15% Mini-PROTEAN® TGX™ gels (Bio-Rad, Hemel Hempstead, UK) and separated by gel electrophoresis at 290 V for 15 min. The gels were then transferred to PVDF membranes (Trans-Blot Turbo Transfer Packs, Bio-Rad) using the Trans-Blot® Turbo™ System (Bio-Rad). Membranes were blocked for 1 h in 5% milk in PBS containing 0.1% v/v Tween® 20 (Sigma-Aldrich) (PBST) and incubated with rabbit anti-human CXCR4 clone UMB2 (1:1000; ab124824, Abcam, Cambridge, UK), Turbo-RFP (1:1000, ab109809, Abcam) or mouse anti-β-actin antibodies (1:10,000, ab6276, Abcam) in 5% milk-TBST overnight at 4°C. After washing with PBST, goat anti-rabbit Immunoglobin G (IgG) horseradish peroxidase (IgG-HRP) (1:2000, SC-2004, Santa Cruz Biotechnology, Dallas, Tx, USA) or goat anti-mouse lgG-HRP (1:2000, SC-2005, Santa Cruz Biotechnology) were incubated in 5% milk-TBST for 1 h at room temperature (RT). Signals were detected using Amersham enhanced chemiluminescence (ECL) Plus Western Blotting Detection Reagent kit (GE Healthcare Life Sciences, Little Chalfont, UK) and Amersham Hyper-film (GE Healthcare Life Sciences). Intensity of protein bands was normalized to β-actin and analyzed using ImageJ version 1.44 h (US National Institutes of Health, Rockville, MD, USA).

### Immunofluorescence

MDA-MB-231 cells (100,000 cells/well) were plated in slide chambers (Nunc® Lab-Tek®, Sigma-Aldrich) and incubated overnight. CXCR4 expression was modulated by treatment with 0.5 μg/mL of DOX for 24 h. Cells were then fixed with 4% formaldehyde in PBS for 15 min at RT. Cells were washed with PBS, 3 x 10 min, and incubated with blocking and permeabilization buffer (PBS + 1% BSA + 0.1% Triton-X100 (Sigma-Aldrich)) for 1 h at RT. Samples were incubated with rabbit anti-CXCR4 UMB2 clone antibody (1:200) in blocking buffer overnight at 4°C. After incubation with primary antibody, cells were washed with PBS, 3 x 10 min, and incubated with Alexa Fluor® 488 goat anti-rabbit IgG secondary antibody (1:400; Molecular Probes™, Thermo Fisher Scientific) in blocking buffer for 1 h at RT protected from light. Cells were washed with PBS, 3 x 10 min, and further incubated with Alexa Fluor® 594 Phalloidin (1:100; Molecular Probes™) for 20 min at RT protected from light. Following another washing step, coverslips were placed on slides using ProLong® gold antifade mounting reagent with 4’-6-diamidino-2-phenylindole (DAPI) (Life Technologies Ltd., Thermo Fisher Scientific). Immunofluorescence imaging was performed using a 40X or 60X UPlanAPO objective lens on an Olympus BX-51 wide-field microscopy UIS2 optical system (Olympus Life Science Europa GMBH, Hamburg, Germany) equipped with a DP70 digital camera. Images were acquired using an Olympus U-RFL-T epifluorescence source and DPController 1.2.1.108 imaging software (Olympus Optical Co. Ltd, Tokyo, Japan) in the red, blue and green channels. Image processing was performed using ImageJ version 1.44 h.

### Animal models

All animal experiments were conducted by licensed investigators under Project License 7008651 approved on 28^th^ July of 2015 by the Animal Welfare Ethical Review Body (AWERB) and in accordance with the National (UK Home Office) Guidance on the Operation of the Animal (Scientific Procedures) Act 1986 (HSMO, London, UK, 1990) and within the guidelines set out by the UK National Cancer Research Institute Committee on Animals in Cancer Research [51] and Arrive Guidelines.

### Tumor models

Tumor models were established in NOD SCID mice (Charles River UK Ltd, Margate, UK). All mice were female, 6–8 weeks old, weight-matched (20±2g) and kept under standard conditions in individually ventilated cages (maximum of 6 animals per cage) with food and water *ad libitum* in a designated SPF laboratory in the containment room of the animal facility. Mice were allowed 7 days to acclimatize before any procedure was carried out. Xenografts were generated by injecting U2932 or SuDHL8 (5x10^6^ cells in 50% Matrigel (Corning, Amsterdam, The Netherlands)) and 50% PBS (100 μl total), respectively, in the lower flank of mice. Mice were anesthetized with 2.5% isoflurane/O_2_ and placed on a heating mat for the duration of procedure (around 10 min) and monitored until fully recovered. Tumor dimensions were measured by caliper and volumes calculated using the ellipsoid formula that is best for estimating tumor mass: $Volume\;({mm}^{3})=\frac{\pi }{6}\times a\times b\times c$ where a, b and c represent 3 orthogonal axes of the tumor. Tumors reached an appropriate volume (100 mm^3^) around 4 weeks post-implantation and mice were randomized into two groups with size-matched tumors: U2932 and SuDHL8 groups (n = 4 per group).

### *In vivo* ultrasound imaging of T140-MB

US imaging sessions of both experimental groups were intercalated to avoid operator biases. The experimental unit in this study was the single animal and since each animal was injected with both NT-MB and T140-MB (at different times), each animal served as its own control for the purposes of reducing animal numbers. For MB injection, mice were anesthetized with 2.5% isoflurane/O_2_, placed on a heating mat and injected with 50 μl of a solution of 1x10^7^ MB/ml of either NT-MB or T140-MB through i.v. injection *via* lateral tail vein cannula. A waiting period of at least 20 min separated each injection to ensure that all MB were cleared from the blood pool. US imaging was performed using a Verasonics research platform (Verasonics, Redmond, WA, USA), together with a L22-14v probe (Verasonics). B-mode plane wave imaging at a slow frame rate (2 Hz) was acquired for 180 s (3 min), with 15 compounded 1-cycle plane wave pulses at angles spanning between -7.5° and 7.5° (1° steps), transmitted at a center frequency of 18 MHz and a mechanical index of 0.06. Animals were culled straight after scanning by cervical dislocation and tumor tissues were excised and part of the tissue was snap-frozen and part was preserved in formalin (10%, neutral-buffered, Sigma-Aldrich) for paraffin embedding.

### Kinetic modelling of ultrasound imaging data

Radiofrequency (RF) data was acquired in real-time and transferred to a workstation for post-processing; all RF data was reconstructed using a GPU-beamformer. The MB kinetics was evaluated by producing a mask to segment the tumor region in the beamformed images. The global mean time intensity curve (TIC) of the MB enhancement within the tumor’s ROI was then computed and fitted to a gamma-variate function–a standard analysis method for bolus kinetics [52]

$$I(t)=At^{\alpha }e^{-\frac{t}{\beta }}$$

Where A, α and β are the governing parameters of the non-linear least-square curve fitting (S1 Fig). This model allows extraction of certain biologically-relevant parameters, such as blood volume, which corresponds to the area under the curve (AUC); the wash-in and wash-out rates of the contrast agent, which are related to the slopes of the time-intensity curve (TIC); the mean transit time (mTT), which is the time for which the AUC is ½ of its total volume, and is essentially related to the amount of time that MB spend, on average, in the tumor region; the peak signal intensity of the curve, which is the maximal contrast enhancement in the ROI; the time of arrival, i.e., the time from bolus injection to the arrival in the ROI, and the time to peak enhancement. For visualization of the tumor vasculature, Acoustic Sub-Aperture Processing (ASAP) technique was used that explores the spatio-temporal coherence of the classical Power Doppler (DP) signal. Briefly, two sets of images were reconstructed by splitting the channels into two non-overlapping sub-apertures. Singular value decomposition (SVD) was applied to the two sets of reconstructed Doppler signals to remove the tissue clutter [53, 54]. Cross-correlation was performed by multiplying the two Doppler signals and averaging them across frames.

### PET imaging

For PET imaging studies, mice were anesthetized with 2.5% isoflurane/O_2_ and placed in a thermostatically controlled ring in a dedicated small animal Genisys^4^ PET scanner. Following injection of 1.48 MBq of [^18^F]MCFB *via* lateral tail vein cannula, 0–60 min post-injection (p.i.) dynamic or 40–60 min p.i. static PET scans were acquired in a list-mode format to give decay-corrected values of radioactivity accumulation in tissues. The collected data were reconstructed with a 3-dimensional maximum likelihood estimation method (3D ML-EM). Volumes of interest (VOIs) of tumors were manually defined using Siemens Inveon Research Workplace software in the summed PET images and used to compute the time-activity curves (TACs). Tumor radioactive uptake was quantified and normalized to average whole-body uptake.

### Immunohistochemistry

For immunohistochemical staining, formalin-fixed paraffin embedded tumor sections were cut into slices of 5 μm thickness. These sections were de-paraffinized in xylene, rehydrated in graded alcohols and heated in a microwave oven at 900 W for 30 min in citrate buffer at pH 6.0. Endogenous peroxidase activity was suppressed before immunostaining with Powerblock reagent (Biogenex, Fremont, CA, USA). Sections were stained with Mayer’s hematoxylin, or incubated with rabbit anti-human CXCR4 clone UMB2 (1:1000) or rabbit anti-human CD31 (1:50, ab28364, Abcam) and with the secondary antibodies Alexa Fluor® 488 goat anti-rabbit IgG (1:400; Molecular Probes™) and Alexa Fluor® 594 goat anti-rabbit IgG (1:400; Molecular Probes™). The slides were mounted using ProLong® Gold Antifade mounting reagent with 4’-6-diamidino-2-phenylindole (DAPI) for cell nuclei staining. Immunofluorescence imaging was performed using a 40X UPlanAPO objective lens on an Olympus BX-51 wide-field microscopy UIS2 optical system (referenced above) in the red, blue and green channels.

### Microvessel density calculation

Microvessel density (MVD) was calculated by counting the blood vessel occurrences per field-of-view (FOV) of the examined tumor specimens, normalized to the number of images counted (n = 6).

### Statistical analysis

Data were expressed as mean ± SEM. Statistical analysis was done using unpaired 2-tailed Student’s t-test or two-way ANOVA with Bonferroni correction as appropriate and defined as significant (* p<0.05), very significant (** p<0.01) and extremely significant (***, p<0.001).

## Results

### Development of a CXCR4-targeting MB

The CXCR4-targeting of the MB was achieved by amide bond formation between the T140 peptide and the amine group of one of the lipid components of the MB shell, DSPE-PEG_2000_-NH_2_, forming the lipo-peg-peptide, DSPE-PEG_2000_-T140 (Fig 1A). Whereas NT-MB’ shell is composed of the lipids DPPC, DPPA and DSPE-PEG_2000_-NH_2_, the targeted MB (T140-MB) were developed by replacing half (4.8%) of the original mole fraction of DSPE-PEG_2000_- NH_2_ in the MB’ shell with the lipo-PEG-peptide, DSPE-PEG_2000_-T140 (S1 and S2 Tables). We investigated whether this modification of the MB’ shell would introduce variability in certain properties such as size and concentration; T140-MB and NT-MB were produced with similar concentrations (9x10^8^ MB/ml and 1x10^9^ MB/ml, respectively—Fig 1B) and size distribution (1.7±0.7 and 2.0±0.9 μm, respectively—Fig 1C), as determined by counting and sizing analysis of the FOVs acquired (Fig 1D), with small oscillations throughout the 3 weeks evaluated. The solutions had a milky appearance both straight after and 15 min post-reconstitution (Fig 1E). Potential cytotoxicity of the contrast agents was assessed by incubating the T140- and NT-MB components with HepG2 cells for 4h. There was no significant cell membrane damage in any of the concentrations tested relative to vehicle (Fig 1F).

### *In vitro* T140-MB binding

**Fig 2:**
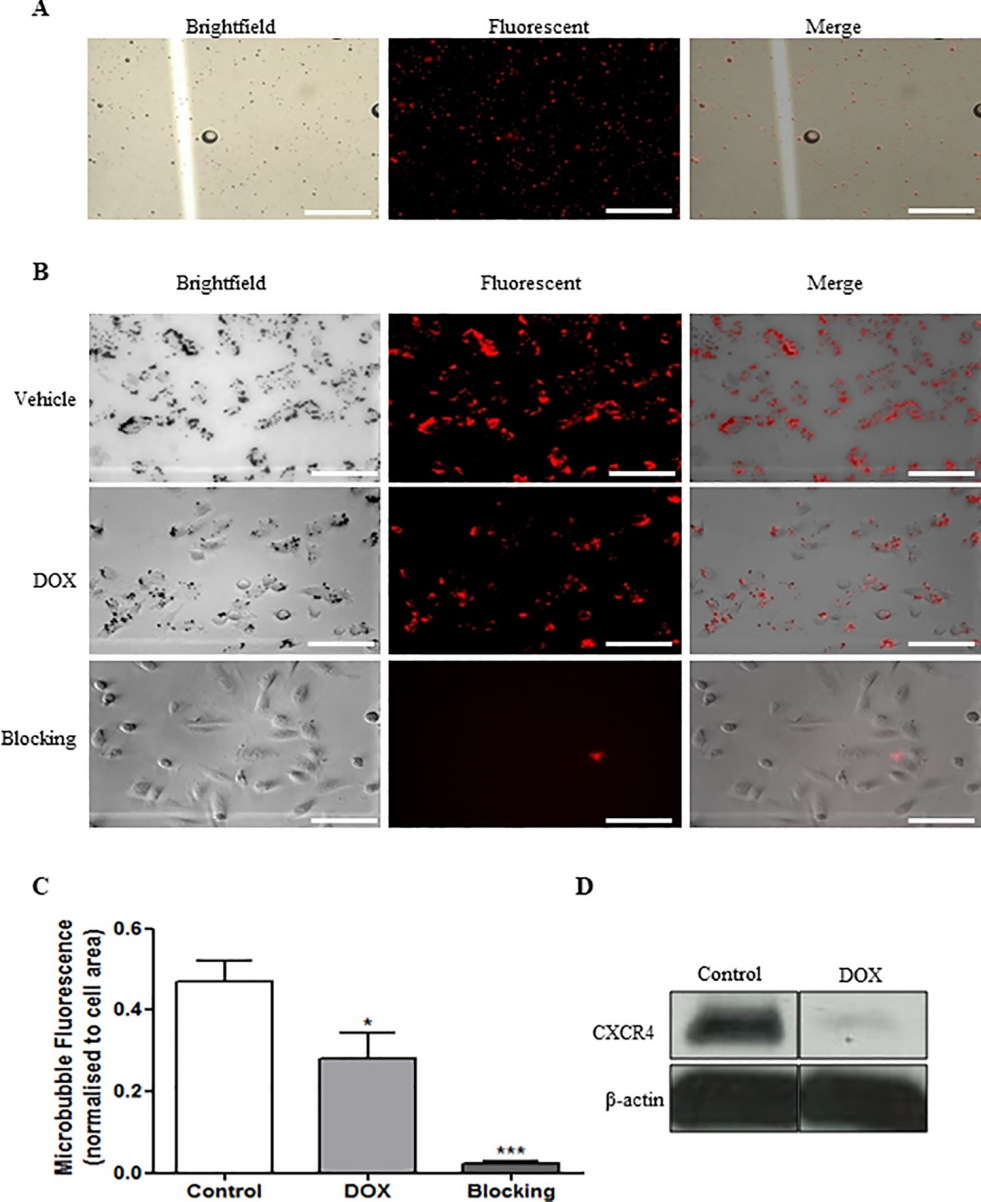
T140-MB binds sensitively and specifically to CXCR4-expressing cells. (A) Brightfield, fluorescent and merged images of T140-MB modified with 2 μg/mL of DiLC_18_(3) and (B), of T140-MB binding to MDA-MB-231 shCXCR4 cells in the presence of vehicle, DOX (0.5 μg/mL for 24h), or blocking with free T140 peptide (1 mg/mL for 5 min). (C) Quantification of microbubble fluorescence normalized to cell density. Data represents mean ± SEM, n = 6. (D) Confirmation of CXCR4 knockdown in the presence of DOX determined by western blot. β-actin was used as loading control. Images were obtained under 200x magnification and scale bar represents 100 μm.

In order to validate the CXCR4-targeting component of T140-MB, we incubated them with isogenic models with varying receptor expression achieved through a DOX-inducible shRNA that causes knockdown of CXCR4. To better distinguish the contrast agents from cells, we fluorescently-labelled T140-MB with Dil, a lipophilic dye that incorporates into the MB membrane (Fig 2A).

T140-MB binding was sensitive to CXCR4 expression, showing significantly less accumulation in DOX-treated MDA-MB-231 shCXCR4 compared to control (60% drop in fluorescence intensity, *p*<0.05, Fig 2B and 2C), proportionally to the drop in CXCR4 expression (77%, Fig 2D). Specificity to CXCR4 was shown by the 95% abrogation of binding through blocking with 1 mg/ml of free T140 5 min prior to T140-MB incubation. This behavior was exclusive to T140-MB while NT-MB did not bind to cells (S2 Fig).

### Ultrasound imaging of vascular CXCR4 with T140-MB

**Fig 3:**
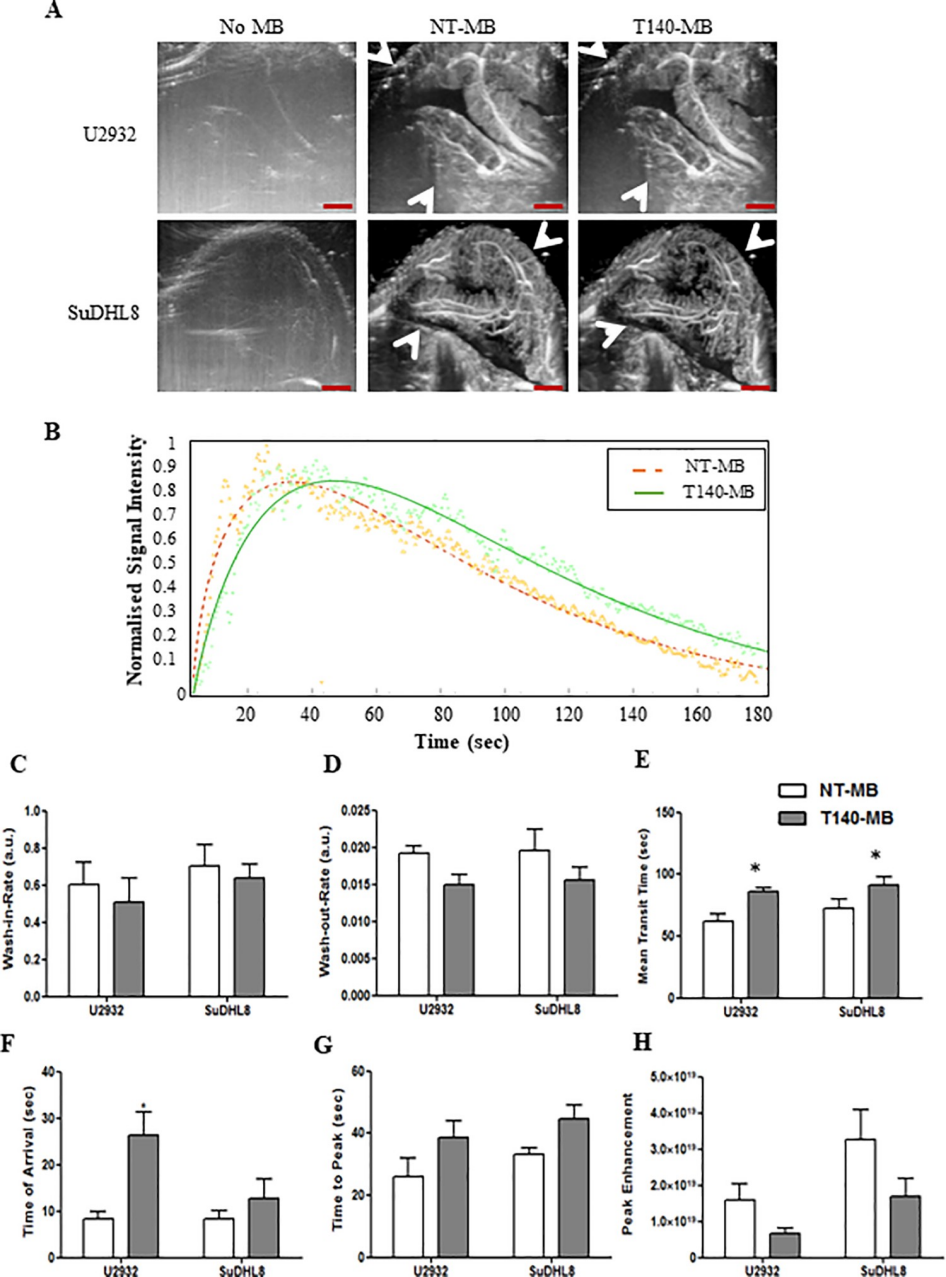
*In vivo* ultrasound imaging of U2932 and SuDHL8 tumors with NT-MB and T140-MB. (A) Representative clutter-filtered Power Doppler images of U2932 and SuDHL8 tumors before and after injection of NT-MB and T140-MB (white arrows indicate tumor borders). Scale bar represents 1 mm. (B) Representative time-intensity curve derived from region of interest analysis in a SuDHL8 tumor, representing T140-MB (green) and NT-MB (orange) kinetics for 3 min after bolus injection. Dots represent the raw data over the 180 sec (2 Hz) and the solid line represents gamma-variate function fit. Perfusion parameters wash-in-rate (C), wash-out-rate (D), mean transit time (E), time of arrival (F), time to peak (G) and peak enhancement (H) were extracted from the time-intensity curves for both MB. Data represents mean ± SEM, n = 6.

To evaluate T140-MB’s ability to image receptor expression *in vivo*, we used two tumor models with reported differential expression of CXCR4, U2932 (high) and SuDHL8 (low) [33, 55]. High frequency, low intensity US was used to image both tumor models before and during perfusion with T140-MB and NT-MB through the tumor’s vasculature. Fig 3A shows representative US images after clutter-filtering for rejection of tissue signal with exceptional spatial resolution, allowing identification of microvessel [56].

Segmentation of the tumor area was carried out in pre-processed US images and time-intensity curves were generated; representative time-intensity curves of T140-MB and NT-MB in a SuDHL8 tumor is shown in Fig 3B, where signal intensity was normalized against the peak intensity to reduce variability associated with the MB injection.

T140-MB showed non-significant slower wash-in (Fig 3C) and wash-out rates (Fig 3D), but persisted for longer periods in the tumor region, with mean transit time difference of 23,6±2,1 sec in U2932 and 19,4 sec in SuDHL8 tumors (p<0.05 Fig 3E), indicating binding of these MB to the vasculature. T140-MB were faster to arrive at the US FOV of the tumors (Fig 3F), particularly in U2932 tumors, which could be hypothesized to be due to possible retention of the T140-MB in other CXCR4-expressing organs; and non-significantly quicker to produce peak contrast enhancement (Fig 3G), although with lower echogenicity than their non-targeted counterparts (Fig 3H). These parameters are unlikely to reflect any variability in the injection procedure, since all injections were concentration-adjusted and timed in the same fashion.

Interestingly, despite the high differences of CXCR4 expression reported in the literature [33, 55], there were no significant differences in the perfusion parameters between the two tumor models used, which may be due to unsuitability of our targeted-MB to provide efficient readouts on CXCR4, or insufficient differences in receptor expression between the vasculature of these models.

In some of the US images, there are dark areas in the center of U2932 tumors, indicative of areas without perfusion due to necrosis, as evident in the H&E images (S3 Fig). This is likely due to a faster tumor growth rate of the U23932 tumor model compared to SuDHL8 (160±24 and 66±1 mm^3^, respectively, 36 days after tumor inoculation, S3 Fig).

### PET imaging of whole-tumor CXCR4 with [^18^F]MCFB

In order to further explore the mechanism behind the lack of differences in the behavior of the targeted-MB in the tumor models used, we validated CXCR4 expression in the same tumor models with [^18^F]MCFB, a CXCR4-targeting small-molecule analogue PET imaging agent previously developed by our group [33].

**Fig 4:**
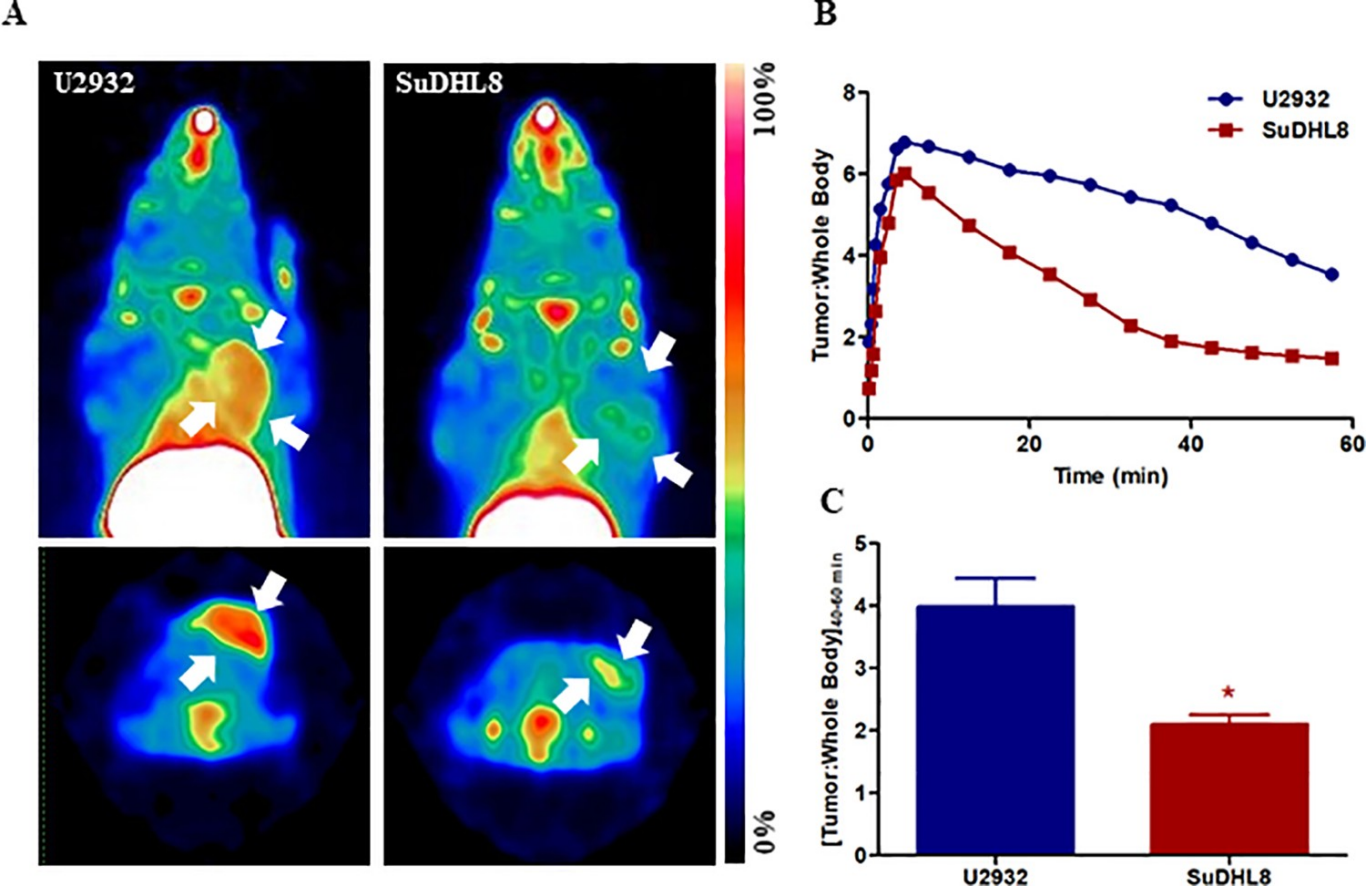
[^18^F]MCFB shows differential uptake in U2932 and SuDHL8 tumors. (A) Representative maximum intensity projection (top) and axial (bottom) PET images derived from 40–60 min (p.i.) static scans of U2932 and SuDHL8 xenograft-bearing mice. White arrows indicate tumors. (B) Time-activity curve derived from ROI analysis of 0–60 min dynamic [^18^F]MCFB-PET and (C) quantification of tumor uptake of [^18^F]MCFB 40–60 p.i., normalized to whole-body uptake. Data represents mean ± SEM, n = 4.

Representative maximum intensity projection (MIP) and axial PET images are shown in Fig 4A, where both U2932 and SuDHL8 tumors are clearly visible at 40–60 min post tracer injection (p.i.). Dynamic PET data of 0–60 min p.i. was used to derive time-activity curves of both tumor models; Fig 4B shows peak uptake of [^18^F]MCFB at 5 min post injection due to first-pass perfusion. Notably, while peak enhancement was comparable for both tumor models, the wash-out in SuDHL8 tumors was more pronounced, with [^18^F]MCFB specific uptake at 40–60 min post-injection considerably higher in U2932 compared to SuDHL8 tumors (4.0±0.4 and 2.1±0.2 tumor to whole body ratios, respectively; p<0.05 Fig 4C). Interestingly, while this is in accordance to our previous studies [33], these differences were not reproduced in the US data. While [^18^F]MCFB is a tumor-penetrating small molecule, capable of providing whole-tumor compartment readouts of CXCR4 expression, T140-MB are micron-size contrast agents that do not extravasate the vasculature within the imaging time window and thus will only detect CXCR4 expression in this compartment. We hypothesized that the incongruence of the imaging data from these two modalities are a reflection of non-linear differential expression of CXCR4 between the vascular and tumor compartments within the same tumor model.

### Analysis of total and vascular CXCR4 expression

To investigate whether the lack of significant differences between tumor models was receptor-mediated or merely related to MB delivery, the total tumor CXCR4 expression, its vascular expression and microvessel density (MVD) were determined through immunohistochemical analysis of excised tumor specimens.

**Fig 5:**
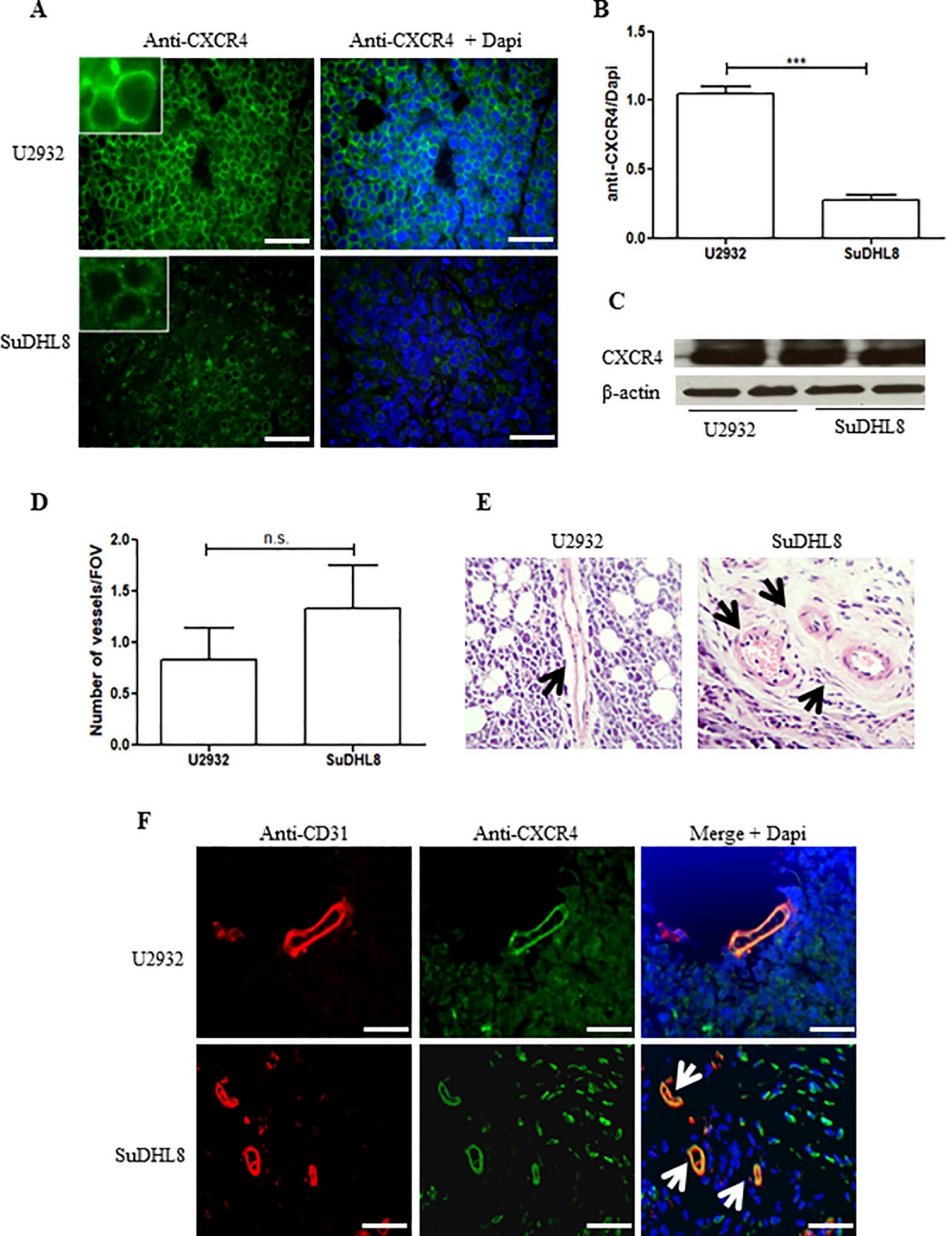
CXCR4 expression and microvessel density in U2932 and SuDHL8 tumors. (A) Representative immunofluorescence images of excised tissues after CXCR4 staining with an anti-CXCR4 antibody, (UMB2) and Dapi for nuclei staining. (B) Quantification of CXCR4 fluorescence normalized to Dapi. (C) CXCR4 expression in the tumor tissues was further confirmed by western blot and β-actin was used as loading control. (D) Microvessel density in U2932 and SuDHL8 tumors was determined by counting the blood vessel occurrences/FOV in the haematoxylin & eosin staining preparations of excised tissues (E). (F) Immunofluorescence images showing blood vessels (anti-CD31 antibody) that co-localize with CXCR4 (anti-CXCR4 antibody, UMB2) in both tumor models. Dapi was used for nuclei staining. White and black arrows indicate blood vessels. Images were obtained under 400x magnification and scale bar represents 200 μm. Data represents mean ± SEM, n = 6.

Fig 5A shows representative immunofluorescence images of U2932 and SuDHL8 tumors, demonstrating high CXCR4 expression in the cell membrane, and, to some extent, in the cytoplasm. Fluorescence quantification showed a 4-fold higher expression of CXCR4 in the U2932 tumors relative to SuDHL8 (*p*<0.001) (Fig 5B); this was further confirmed by western blot analysis of tumor lysates (Fig 5C). Of note, the apparent absence of CXCR4 expression in the western is due to the intensity thresholding applied to avoid oversaturation of the remaining bands. These results provide a correlation between the PET imaging data and CXCR4 expression, but do not help explain the US data; a more relevant parameter is the degree of vascularization and the CXCR4 expression in the vasculature. Microvessel density (MVD) (Fig 5D) was determined by counting the blood vessel occurrences per FOV depicted in the haematoxylin & eosin or anti-CD31 staining preparations; CD31 is constitutively expressed on vascular endothelial cells and thus widely used as a marker to demonstrate the presence of microvessels [56].

Interestingly, quantification of the degree of vascularization showed that microvessel density is similar between the two models (0.8±0.3 microvessels/FOV in U2932 and 1.3±0.4 in SuDHL8 tumors, Fig 5F). More importantly, CXCR4 staining in the same preparations showed co-localization with CD31 staining with similar intensity for both SuDHL8 and U2932, indicating that the marked differences of CXCR4 expression in the tumor cells are not reflected in the respective tumor vasculatures.

## Discussion

In this study we report, for the first time, non-invasive US imaging of CXCR4 expression in the tumor vasculature using a CXCR4-targeted contrast agent, T140-MB. Through comparison with the tumor-penetrating [^18^F]MCFB PET agent, we found that, interestingly, whole-tumor CXCR4 does not necessarily reflect the receptor’s expression in the tumor vasculature.

One very important consideration in the design of a functionalized MB is the targeting ligand. Ideally, it should be therapeutically relevant, bind to its target with high selectivity and show low levels of non-specific accumulation. T140 peptide analogues are strong CXCR4 antagonists and are being investigated clinically, having entered Phase 3 clinical trials (BL-8040 analogue, *ClinicalTrials*.*gov Identifier*: *NCT03246529*) in 2018 as a combination treatment for multiple myeloma. While imaging studies with CXCR4-targeting molecules often result in unwanted tracer accumulation in metabolic organs such as the liver and kidneys, the T140 peptide has been shown to have favorable biodistribution [39] relative to other known molecules [34, 37].

The immunogenicity of the MB was not determined in this work but low immune reactions have been reported for similar formulations [57, 58] and only after repeated administration. Furthermore, the use of covalent coupling rather than (strept)avidin/biotin conjugation for MB functionalization further decreases potential immunogenicity [59].

The targeting heterolipid DSPE-PEG_2000_-T140 was incorporated in the MB successfully and without compromising their formation or optical characteristics. These MB were stable for the period tested (3 weeks) when kept as lyophilized lipid components in sealed vials. These vials could be stored at 4°C and easily reconstituted before use, making these contrast agents easy to store and convenient for widespread use. In the T140-MB, 4.8% of DSPE-PEG_2000_-NH_2_ was replaced by the lipo-peg-peptide DSPE-PEG_2000_-T140; it is important to note that only a percentage of the total shell components is incorporated into the bubble’s shell, but the relative ratio is expected to remain the same [60]. Although the number of ligands per MB was not determined in this work, the percentage of modified lipid was sufficient for binding of T140-MB to CXCR4-expressing cells in *vitro*. By using a CXCR4 inducible knockdown MDA-MB-231 cell line, we were able to investigate the effect of the receptor density on the MB binding whilst minimizing the sources of variability that may be introduced by using non-isogenic models. T140-MB bound sensitively and specifically to CXCR4, as demonstrated by the decrease or complete abrogation of MB binding that was concomitant with variations in receptor expression or competitive binding. These MB were found to be non-toxic to cells.

To evaluate these contrast agents *in vivo*, we used two human lymphoma tumor models, U2932 and SuDHL8, that have been reported to be high and low in CXCR4 expression, respectively [33, 55]. High frequency, low intensity US was used to image perfusion of MB through the tumor vasculature with exceptional spatial resolution, and a gamma variate function—a standard analysis method for bolus kinetics [52]–was fitted to the data, allowing extraction of relevant features. A slower wash-out rate combined with significantly longer mean transit time suggests that T140-MB is transiently accumulating in the tumor’s vasculature. Notably, the differences between NT-MB and T140-MB are only modest, but close inspection of the literature showed similar effect sizes with the use of other targeted MB, including BR55, a VEGFR2-targeted MB that is now undergoing clinical trials [61]. Also of note, comparison with NT-MB is often not performed [62, 63]. It is possible that the short period of time used to image these contrast agents (3 min post-injection only) is not sufficient to distinguish free-flowing from bound MB, and that longer acquisition periods are needed. In fact, Tardy *et al*. found that, in prostate tumors, only at the late phase enhancement stage (>200 sec p.i.) did the kinetics of BR55 and SonoVue differ: BR55 show a strong residual signal in the tumor compared to SonoVue, and this was particularly obvious when circulating MB were no longer detectable [64]. Similarly, a different study using a neuropilin-1-targeted MB showed identical tumor accumulation compared to non-targeted counterparts at 4 min p.i., with differences due to MB attachment only visible as late as 8 min post-injection [65]. In this work, we prioritised higher frame-rates over longer acquisition periods. Although it is not completely understood whether our MB generates signal at these later time points, future work will focus on longer acquisitions, whilst preserving sufficient frame-rate for accurate tracking of MB kinetics.

Interestingly, we could not distinguish between the two tumors used, U2932 and SuDHL8, despite the differential CXCR4 expression between these two models that has been described by us [33] and other groups [55]. In order to investigate the mechanism responsible for the lack of significant differences between these two models, we used a PET tracer developed by our group, [^18^F]MCFB; this tracer is a fluorine-18 labelled small molecule analogue of the CXCR4-targeting AMD3465, that due to its small size can extravasate the tumor vasculature and penetrate all tumor compartments, providing readouts of whole-tumor CXCR4 expression. Dynamic kinetic analysis of [^18^F]MCFB showed rapid (within 5 min) increase in tumor signal, with both tumors showing comparable peak enhancement which is indicative of similar initial tracer delivery. Over time however, and as expected, [^18^F]MCFB wash-out kinetics differed in the two models due to specific target binding, with U2932 tumor uptake being 2-fold higher than that of SuDHL8 at 40–60 min post-injection. Analysis of CXCR4 expression on excised tumor tissues corroborated the large differences in receptor expression between the two models. Interestingly, however, was the discovery that these tumors are equally vascularized: MVD analysis showed similar occurrences of blood vessels per FOV. And more interestingly even, was the fact that the vasculature of both tumor models expressed similar amounts of CXCR4. Thus, the lack of differences obtained with the T140-MB is a reflection of the similarities between vascular density and expression of CXCR4 between U2932 and SuDHL8. Our data seem to indicate that receptor expression in the tumor vasculature may be independent from cellular expression. While CXCR4 expression and its prognostic value has been far better characterized in the tumor interstitium than in its vasculature, it is possible that vascular CXCR4 may be a standalone biomarker of tumor development. For example, expression of CXCR4 in the vasculature of hepatocellular carcinoma has been correlated with poor prognosis [66], whereas CXCR4-positive microvessels were significantly associated with tumor growth and UICC stages in gastric [67] and colorectal cancer [68].

Conclusions concerning the prognostic value of vascular CXCR4 in our tumor models are challenging. Despite the equivalent MVD and vascular CXCR4, U2932 are faster-growing tumors compared to SuDHL8. However, it is important to note that while the amount of total tumor vascularization must increase rapidly in fast-growing tumors to support increasing metabolic demands, the density of vessels need not be high; thus, MVD is not a direct indicator of tumor’s growth rate [69]. An important tumor adaptation involves lower rate of oxygen consumption, as well as superior ability to withstand oxygen and nutrient deprivation, which precludes the need of proximity to the vasculature [70, 71]. Whereas some studies have associated increasing MVD to higher histological malignancy grade, poorer prognosis and patient survival in patients with malignant lymphomas [72–74], other studies found no such correlation [75].

Perhaps these data are an indication that tumorigenic and metastatic functions are distinct. In fact, it has been shown that, in the invasive front of pancreatic tumors, the depletion of a distinct subpopulation of CD133^+^ and CXCR4^+^ cancer stem cells significantly reduced the metastatic phenotype without affecting tumorigenesis [76]. It is possible that both U2932 and SuDHL8 models are equally metastatic in relation to vascular CXCR4 dominantly affecting invasion and extravasation, but differently regulated by other factors that promote tumor growth. SuDHL8 and U2932 are DLBCL subtypes with the former being GCB (germinal canter) [77] and latter being ABC (activated B-cell like) [78], with ABC being the least curable subtype [79]. It would be interesting to further evaluate the clinical value of vascular CXCR4, perhaps by determining both tumor cell and vascular CXCR4 expression (co-localised with CD31 for indication of vasculature) and correlating this with other malignancy processes, such as angiogenesis, metastatic potential, and, at later stages, patient survival.

While our work aimed to develop quicker, bedside-friendly means to detect CXCR4 expression in tumors through the development of easy-to-use T140-MB-US imaging methodology, our data ended up providing insights about the relationship of receptor expression between the tumor cell and vascular compartments. Furthermore, it has brought attention to the possible clinical value of vascular CXCR4 as an independent prognostic marker.

## Conclusions

In this work, we successfully developed a T140-MB that can be used for US imaging of CXCR4 expression in the vasculature of cancer. Notably, we also discovered that the presence of CXCR4 in cancer cells does not reflect protein expression in the vascular compartment.

## Supporting information

S1 Fig Example of MB kinetics profile in a tumour obtained by US imaging. The Power Doppler, clutter-filtered images were used to segment the tumour and the global mean time intensity curve (TIC) of the MB enhancement was used to fit a gamma-variate function. This model enables extraction of biologically relevant parameters such as wash-in and wash-out rates, time of arrival, peak enhancement and time to peak, and the mean transit time that corresponds to the time for which the area under the curve is ½ of its total value.
(DOCX)

S2 Fig Qualitative comparison of *in vitro* binding of NT-MB and T140-MB to CXCR4-expressing cells. (A) Brightfield images of T140-MB and NT-MB binding to MDA-MB-231 cells transduced with a non-coding (shSC) or a CXCR4-targeted shRNA (shCXCR4) in the presence of vehicle, DOX (0.5 μg/mL for 24h), or blocking with free T140 peptide (1 mg/mL for 5 min). Images were obtained under 400x magnification and scale bar represents 100 μm.
(DOCX)

S3 Fig Growth and necrosis in tumours. (A) Ultrasound images show a dark region without perfusion, indicating some degree of necrosis, which was confirmed by haematoxylin and eosin staining shown in (B). (C) Growth curve of U2932 and SuDHL8 tumours determined by regular measurements of volume by callipers. Arrows indicate tumour edges and scale bar represents 1 mm in (A) and 100 μm in (B).
(DOCX)

S1 Table Constituents of NT-MB formulation. (DOCX)

S2 Table Constituents of T140-MB formulation. (DOCX)

S1 Raw images (PDF)
