# Supplementary material for: Investigating CXCR4 expression of tumor cells and the vascular compartment: A multimodal approach
Source: PLoS One. 2021 Nov 18;16(11):e0260186. doi: 10.1371/journal.pone.0260186 (PMC8601444; doi:10.1371/journal.pone.0260186)
Supplement: S1 Fig — The Power Doppler, clutter-filtered images were used to segment the tumour and the global mean time intensity curve (TIC) of the MB enhancement was used to fit a gamma-variate function. This model enables extraction of biologically relevant parameters such as wash-in and wash-out rates, time of arrival, peak enhancement and time to peak, and the mean transit time that corresponds to the time for which the area under the curve is ½ of its total value. (DOCX) [file pone.0260186.s001.docx]

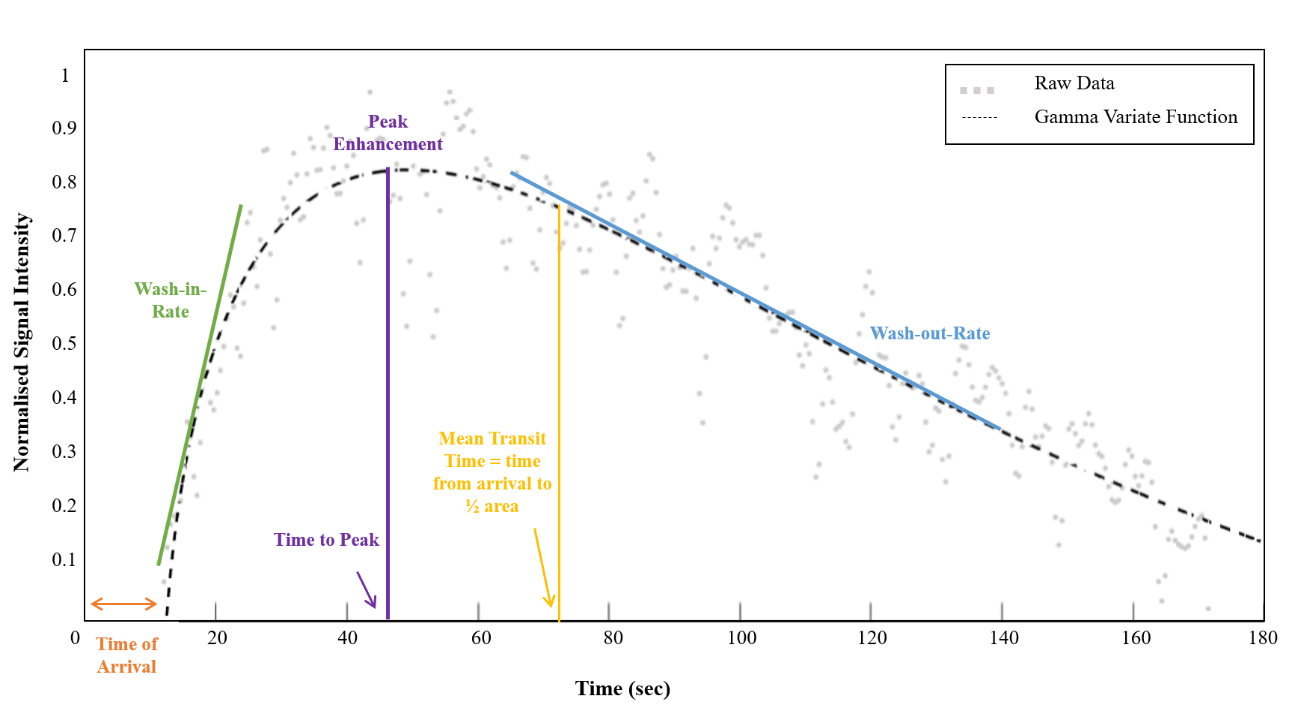


**S1 Fig. Example of MB kinetics profile in a tumour obtained by US imaging.** The Power Doppler, clutter-filtered images were used to segment the tumour and the global mean time intensity curve (TIC) of the MB enhancement was used to fit a gamma-variate function. This model enables extraction of biologically relevant parameters such as wash-in and wash-out rates, time of arrival, peak enhancement and time to peak, and the mean transit time that corresponds to the time for which the area under the curve is ½ of its total value.
