# Supplementary material for: Investigating CXCR4 expression of tumor cells and the vascular compartment: A multimodal approach
Source: PLoS One. 2021 Nov 18;16(11):e0260186. doi: 10.1371/journal.pone.0260186 (PMC8601444; doi:10.1371/journal.pone.0260186)
Supplement: S2 Fig — (A) Brightfield images of T140-MB and NT-MB binding to MDA-MB-231 cells transduced with a non-coding (shSC) or a CXCR4-targeted shRNA (shCXCR4) in the presence of vehicle, DOX (0.5 μg/mL for 24h), or blocking with free T140 peptide (1 mg/mL for 5 min). Images were obtained under 400x magnification and scale bar represents 100 μm. (DOCX) [file pone.0260186.s002.docx]

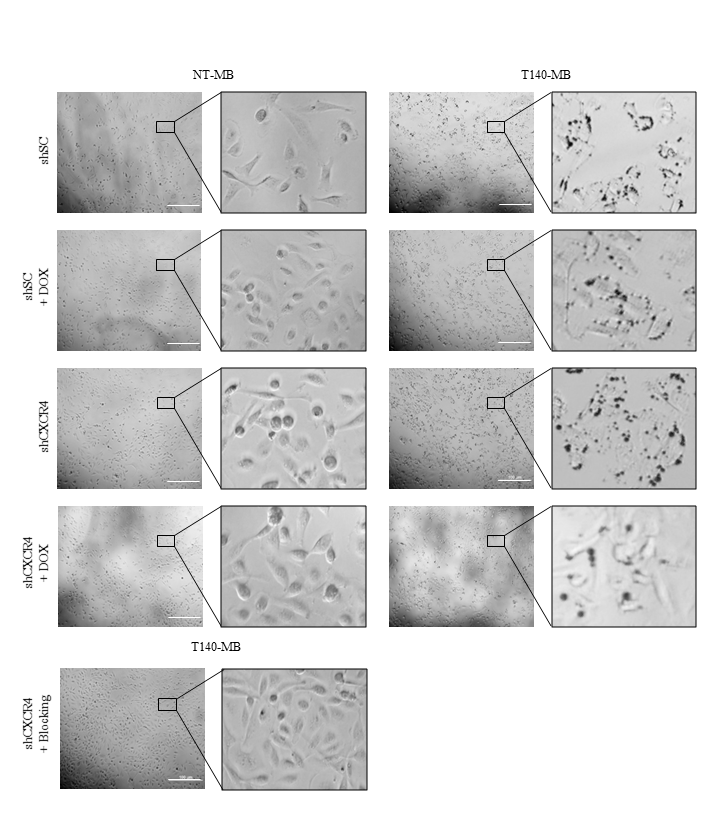


**S2 Fig. Qualitative comparison of *in vitro* binding of NT-MB and T140-MB to CXCR4-expressing cells.** (A) Brightfield images of T140-MB and NT-MB binding to MDA-MB-231 cells transduced with a non-coding (shSC) or a CXCR4-targeted shRNA (shCXCR4) in the presence of vehicle, DOX (0.5 µg/mL for 24h), or blocking with free T140 peptide (1 mg/mL for 5 min). Images were obtained under 400x magnification and scale bar represents 100 µm.
