# Supplementary material for: Investigating CXCR4 expression of tumor cells and the vascular compartment: A multimodal approach
Source: PLoS One. 2021 Nov 18;16(11):e0260186. doi: 10.1371/journal.pone.0260186 (PMC8601444; doi:10.1371/journal.pone.0260186)
Supplement: S3 Fig — (A) Ultrasound images show a dark region without perfusion, indicating some degree of necrosis, which was confirmed by haematoxylin and eosin staining shown in (B). (C) Growth curve of U2932 and SuDHL8 tumours determined by regular measurements of volume by callipers. Arrows indicate tumour edges and scale bar represents 1 mm in (A) and 100 μm in (B). (DOCX) [file pone.0260186.s003.docx]

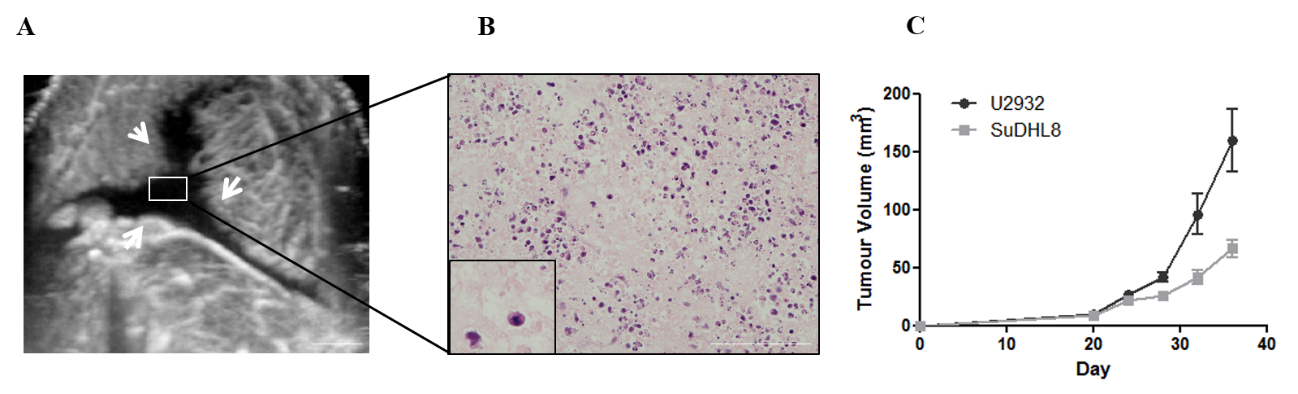


**S3 Fig. Growth and necrosis in tumours.** (A) Ultrasound images show a dark region without perfusion, indicating some degree of necrosis, which was confirmed by haematoxylin and eosin staining shown in (B). (C) Growth curve of U2932 and SuDHL8 tumours determined by regular measurements of volume by callipers. Arrows indicate tumour edges and scale bar represents 1 mm in (A) and 100 µm in (B).
