# Supplementary figures and images for: Investigating CXCR4 expression of tumor cells and the vascular compartment: A multimodal approach
Source: PLoS One. 2021 Nov 18;16(11):e0260186. doi: 10.1371/journal.pone.0260186 (PMC8601444; doi:10.1371/journal.pone.0260186)

**S1 Table - Constituents of NT-MB formulation**

**
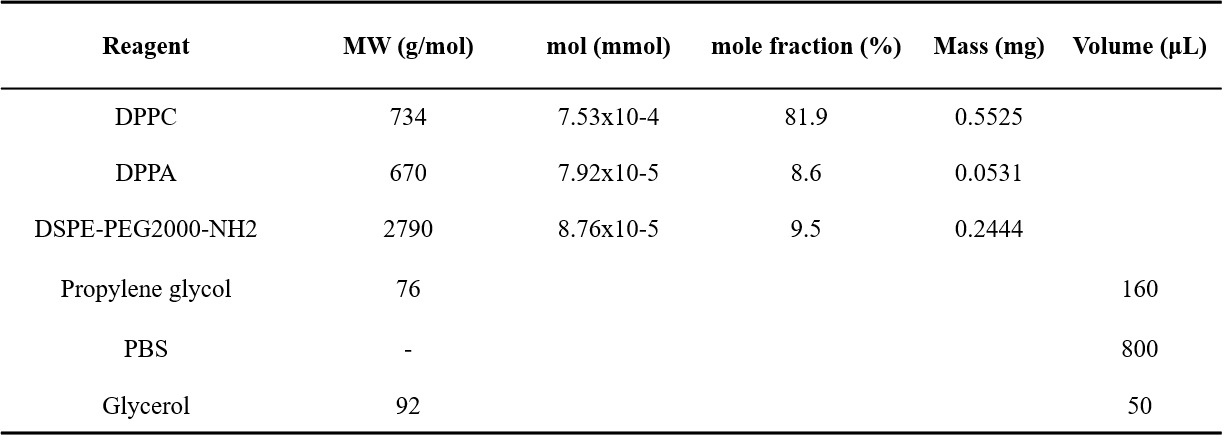
**

Supplement: S1 Table — (DOCX) [file pone.0260186.s004.docx]

**S2 Table - Constituents of T140-MB formulation**

**
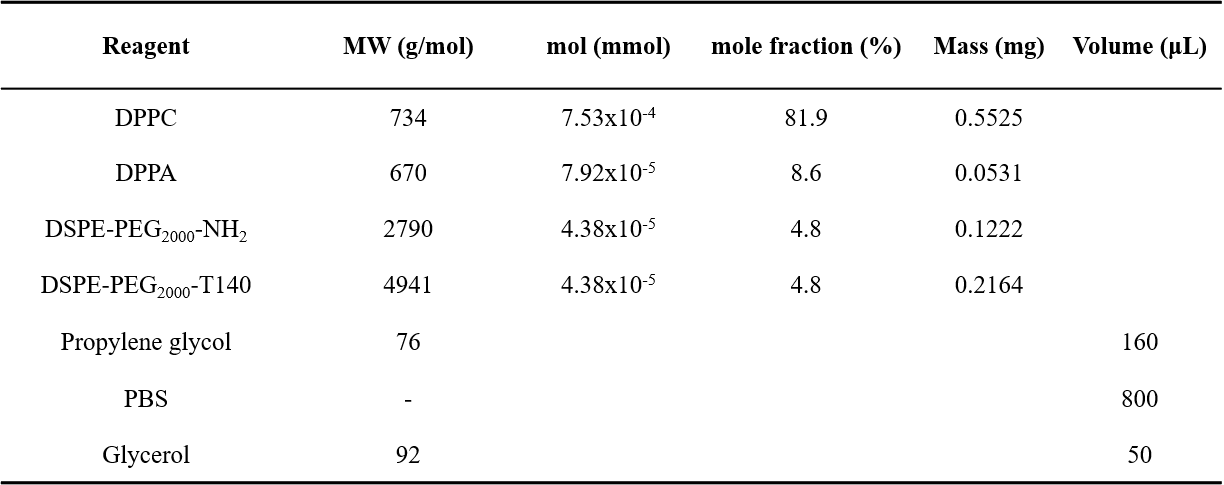
**

Supplement: S2 Table — (DOCX) [file pone.0260186.s005.docx]
