# Supplementary material for: Investigating CXCR4 expression of tumor cells and the vascular compartment: A multimodal approach
Source: PLoS One. 2021 Nov 18;16(11):e0260186. doi: 10.1371/journal.pone.0260186 (PMC8601444; doi:10.1371/journal.pone.0260186)

Original Blots

PONE-D-21-23749 Manuscript

Original Blot from western results reported on Figure 1D:

CXCR4 expression

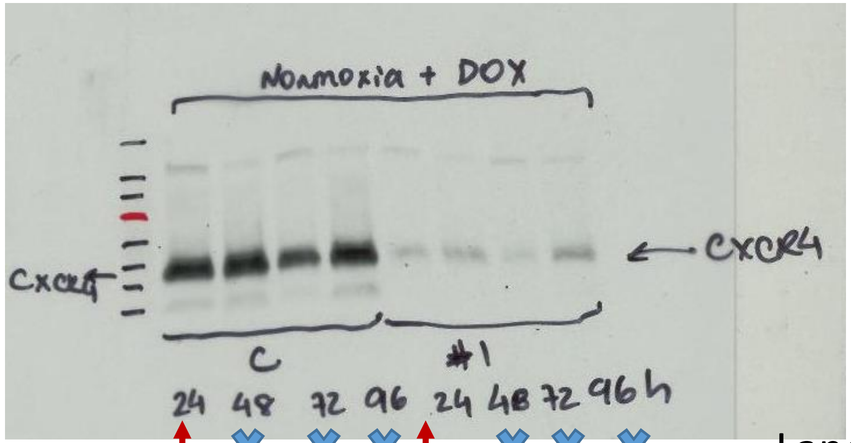

C – Control (vehicle) 24h

#1 – Doxyxycline dosing at concentration 1 (0.5 µg/ml)

Lanes not presented

Lanes presented in Figure 1D

β-actin expression

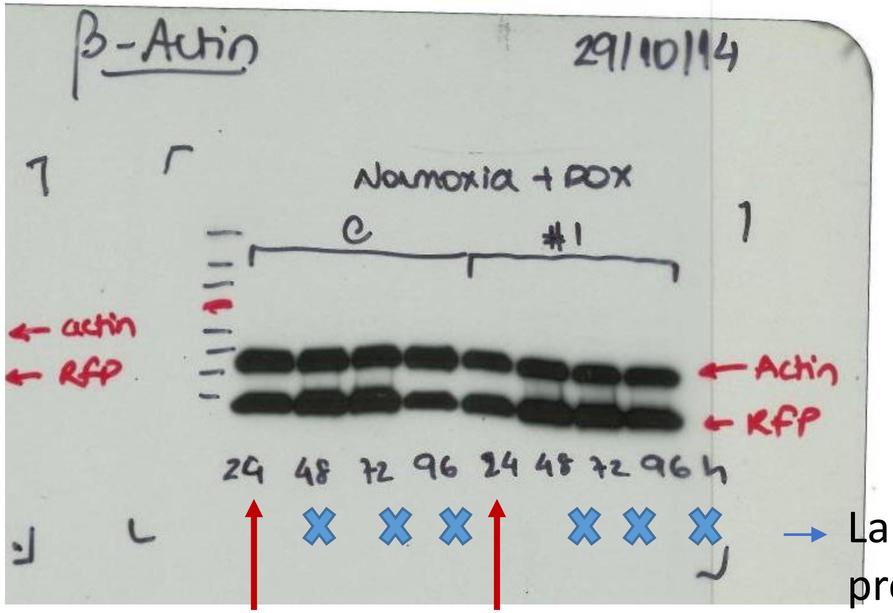

C – Control (vehicle) 24h

#1 – Doxyxycline dosing at concentration 1 (0.5 µg/ml)

Lanes not presented

Lanes presented in Figure 1D

Original Blot from western results reported on Figure 3C:

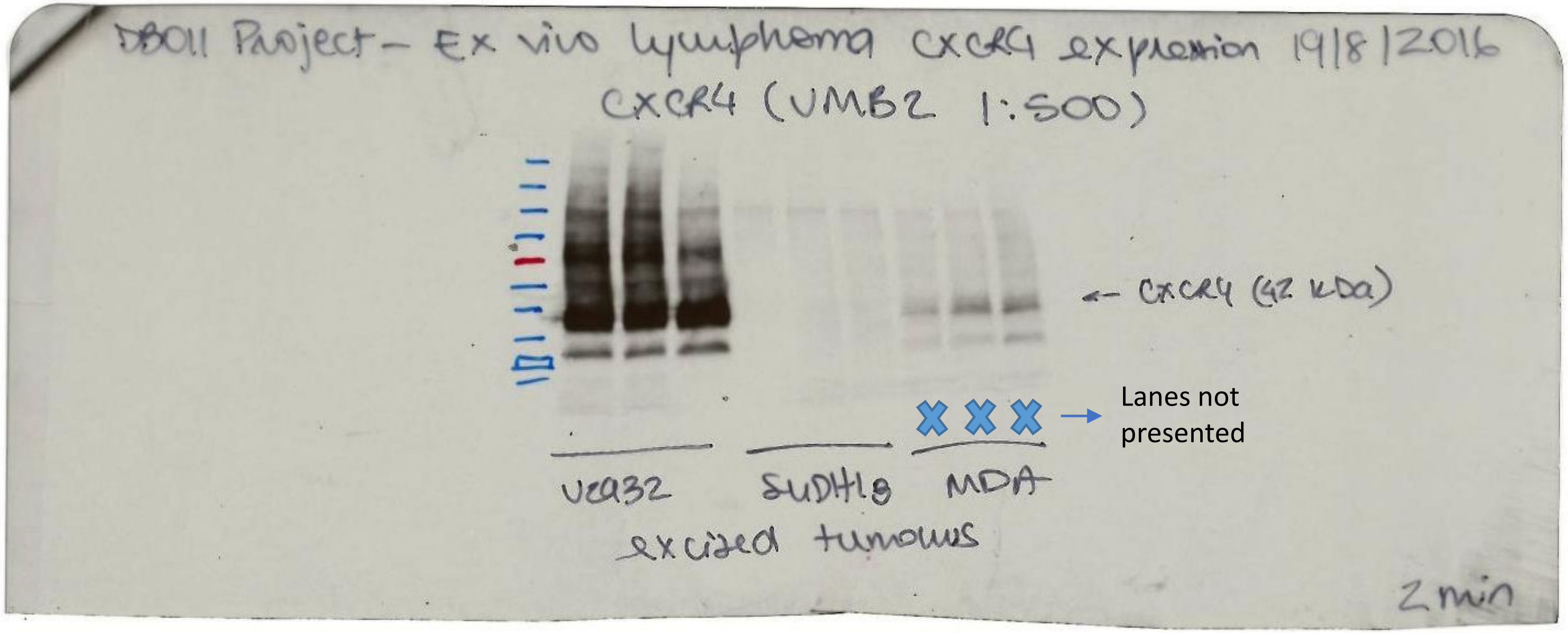

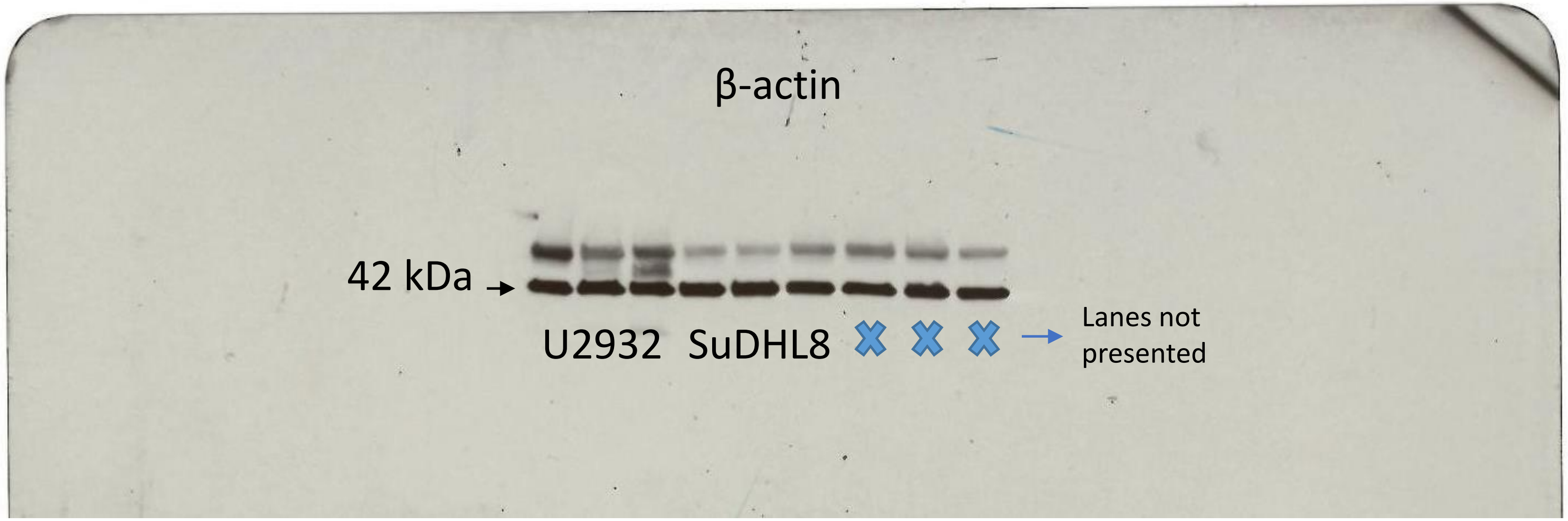

Supplement: S1 Raw images — (PDF) [file pone.0260186.s006.pdf]
